# Peri-Transfer Glucocorticoid Therapy in Oocyte-Donation IVF Bridging the Immunological Gap

**DOI:** 10.3390/ijms27031217

**Published:** 2026-01-26

**Authors:** Charalampos Voros, Fotios Chatzinikolaou, Georgios Papadimas, Spyridon Polykalas, Despoina Mavrogianni, Aristotelis-Marios Koulakmanidis, Diamantis Athanasiou, Vasiliki Kanaka, Kyriakos Bananis, Antonia Athanasiou, Aikaterini Athanasiou, Ioannis Papapanagiotou, Charalampos Tsimpoukelis, Athanasios Karpouzos, Maria Anastasia Daskalaki, Nikolaos Kanakas, Marianna Theodora, Nikolaos Thomakos, Panagiotis Antsaklis, Dimitrios Loutradis, Georgios Daskalakis

**Affiliations:** 1 1st Department of Obstetrics and Gynecology, ‘Alexandra’ General Hospital, National and Kapodistrian University of Athens, 80 Vasilissis Sofias Avenue, 11528 Athens, Greece; depy.mavrogianni@yahoo.com (D.M.); aristoteliskoulak@gmail.com (A.-M.K.); vasilikikanaka@outlook.com (V.K.); tsimpoukelischa@gmail.com (C.T.); thanoskarpouzosdr@hotmail.com (A.K.); md181341@students.euc.ac.cy (M.A.D.); martheodr@gmail.com (M.T.); thomakir@hotmail.com (N.T.); panosant@gmail.com (P.A.); gdaskalakis@yahoo.com (G.D.); 2 Laboratory of Forensic Medicine and Toxicology, School of Medicine, Aristotle University of Thessaloniki, 54124 Athens, Greece; loutradi@otenet.gr; 3 Athens Medical School, National and Kapodistrian University of Athens, 15772 Athens, Greece; dr.georgepapadimas@gmail.com (G.P.); spyridonpolykalas@hotmail.com (S.P.); gpapamd@hotmail.com (I.P.); 4 King’s College Hospitals NHS Foundation Trust, London SE5 9RS, UK; diamathan16@gmail.com (D.A.); kyriakos.bananis@nhs.net (K.B.); 5 IVF Athens Reproduction Center, 15123 Maroussi, Greece; antoathan16@gmail.com (A.A.); diamathan17@gmail.com (A.A.); 6 School of Medicine, European University Cyprus, Nicosia 2404, Cyprus; nkanakas@icloud.com; 7 Fertility Institute-Assisted Reproduction Unit, Paster 15, 11528 Athens, Greece

**Keywords:** infertility, oxidative stress, ovarian ageing, antioxidants, IVF, reproductive endocrinology, molecular research, glucocorticoid, oocyte donation, embryo transfer

## Abstract

In vitro fertilisation via oocyte donation is a unique reproductive technique in which the embryo is fully separate from the receiver. This compels the immune system to exert more effort at the interface between the uterus and the remainder of the body. This setting has maintained interest in peri-transfer glucocorticoid treatment as a possible approach to modify endometrial immunity and enhance implantation. Nevertheless, the data for this procedure are disjointed and mostly derive from investigations on autologous in vitro fertilisation. This narrative review consolidates contemporary evidence on endometrial immunology in oocyte donation cycles, analysing the mechanistic basis, clinical results, and constraints related to peri-implantation glucocorticoid therapy. Outcomes from randomised studies in autologous cycles consistently demonstrate that there is no advantage in live birth rates, but the claimed improvements in clinical pregnancy rates are from heterogeneous and low-quality data. Limited research exists on people who have received oocyte donations. The majority are diminutive and non-random, often integrating glucocorticoids with other therapies such as antibiotics, granulocyte colony-stimulating factor, or endometrial damage. These designs inhibit the dissociation of the independent impact of glucocorticoids. Recent comprehensive randomised studies on recurrent implantation failure further demonstrate the lack of advantages in live births and highlight possible safety issues. The current data do not support the usual use of peri-transfer glucocorticoids in oocyte donation for in vitro fertilisation; nevertheless, short-term, low-dose treatment may be justified in meticulously chosen immunological profiles. There is an urgent need for rigorously designed randomised studies focused only on oocyte-donation recipients to elucidate the therapeutic effectiveness, safety, and suitable clinical context for glucocorticoid treatment in this expanding patient demographic.

## 1. Introduction

The primary factors contributing to the increasing prevalence of in vitro fertilisation by oocyte donation in assisted reproduction are the postponement of childbearing and the decline in a woman’s ovarian reserve with advancing age. In this case, the embryo is completely different from the person who receives it. This makes the immune system work harder where the uterus meets the rest of the body [1]. Peri-transfer glucocorticoid therapy remains a subject of interest in this context as a potential approach to modify endometrial immunity and enhance implantation. However, the data for this process are incomplete and mostly come from studies on autologous in vitro fertilisation [2]. This narrative review examines the mechanistic foundations, clinical results, and constraints of peri-implantation glucocorticoid therapy to aggregate the latest findings on endometrial immunology in oocyte donation cycles. The reported rises in clinical pregnancy rates are founded on inconsistent and substandard data. Outcomes from randomised trials in autologous cycles consistently indicate no advantage in live birth rates. There is limited research on individuals who receive oocyte donations. Most of the studies are small and not random. They often use glucocorticoids, along with other treatments like endometrial damage, granulocyte colony-stimulating factor, or antibiotics. These designs stop glucocorticoids from having separate effects. Recent comprehensive randomised studies on recurrent implantation failure further demonstrate the absence of benefits in live births, while also indicating possible safety concerns. The current data do not support the routine use of peri-transfer glucocorticoids in oocyte donation for in vitro fertilisation. However, short-term, low-dose treatment may be justified in meticulously selected immunological profiles. To elucidate the therapeutic efficacy, safety, and suitable clinical context for glucocorticoid treatment in this expanding patient demographic, meticulously designed randomised studies focussing solely on oocyte donation recipients are urgently required.

### 1.1. The Allogeneic Embryo–Endometrium Interface in Oocyte-Donation Cycles

The implantation environment in oocyte-donation IVF significantly differs from that in autologous cycles, since the embryo is entirely allogeneic to the receiver. The entire genetic divergence of the embryo, which displays a wide range of foreign antigens, requires adaptation by maternal cells, leading to a much improved immunological interface [3]. Human leukocyte antigen (HLA) molecules, such as HLA-C, HLA-G, and HLA-E, display both polymorphism and non-polymorphic traits on the trophoblast surface. These molecules engage with many immunoregulatory mechanisms in the decidua [4].

The interaction between maternal killer immunoglobulin-like receptors (KIRs), mostly located on uterine natural killer (uNK) cells, and extravillous trophoblast HLA-C constitutes a vital molecular roadblock [5]. Once considered innate effectors, uNK cells are now acknowledged as specialised tissue-resident lymphocytes that modulate trophoblast invasion, release angiogenic factors (VEGF, PlGF), and coordinate spiral artery remodelling [6]. The binding of KIR-HLA-C regulates downstream activation or inhibition via the DAP12 (activating) or DAP10 (inhibitory) adaptor systems. Inhibitory KIR/HLA-C2 combinations may result in increased interferon-γ and tumour necrosis factor-α production, leading to insufficient vascular remodelling, superficial invasion, and implantation failure [7]. In contrast, several activating KIR genotypes, including KIR2DS1, facilitate tolerance and control trophoblast invasion. In oocyte donation cycles, when the foetal HLA-C alleles are solely derived from the donor, mismatched KIR–HLA patterns occur with greater frequency.

Decidual dendritic cells and macrophages, as antigen-presenting cells, regulate immunological checkpoint molecules (PD-L1/PD-1, CTLA-4) and co-stimulatory pathways (CD80/CD86, CD40). Dendritic cells may elevate the levels of PD-L1 and indoleamine 2,3-dioxygenase (IDO1) in the presence of an allogeneic embryo. This would initiate the degradation of tryptophan and inhibit T-cell proliferation [8]. However, modified dendritic-cell maturation or excessive pro-inflammatory signalling may circumvent tolerance mechanisms. This may hinder communication between trophoblasts and decidua, hence promoting Th1-dominant CD4+ T-cell development. Maintaining equilibrium between pro-inflammatory Th1/Th17 subsets and regulatory T-cells (Tregs) is crucial. Tregs often proliferate after implantation due to TGF-β and FOXP3 signalling [9]. Research on donor egg cycles reveals that certain recipients have reduced Treg recruitment, leading to a microenvironment skewed towards Th1/Th17-type inflammation and inadequate synthesis of TGF-β and IL-10 [10].

The allogeneic embryo interacts with a progesterone-primed endometrial matrix undergoing significant stromal biological remodelling. Decidual stromal cells (DSCs) produce receptivity mediators, including LIF, IGFBP-1, prostaglandin E2, and integrins such as αvβ3, in reaction to alloantigen exposure by coordinating hormonal signals with immunological responses via the cAMP–PKA, FOXO1, GATA2, and NR2F2 pathways [11]. The activation of allostimulus-dependent nuclear factor κB (NF-κB) may disrupt this equilibrium. This diminishes the expression of HOXA10 and HOXA11 and alters the profiles of adhesion molecules that are crucial for blastocyst attachment [12]. Transcriptomic investigations of donor egg endometria reveal the heightened activity of complement components (C1q, C3), chemokines (CXCL9/10/11), and pattern-recognition signalling molecules.

Recent single-cell RNA sequencing investigations provide more insights: Decidual immune compartments exhibit increased populations of activated uNK subsets and macrophages characterised by M1-like transcriptional profiles, while trophoblast subpopulations in oocyte donation pregnancies show modified expression of HLA-G isoforms, metalloproteinases (MMP2, MMP9), and angiogenic regulators [13]. These data support the concept that donor egg implantation involves a more intricate and perhaps more inflammatory molecular network, necessitating careful coordination of immunological tolerance systems.

### 1.2. Innate Inflammation, Stromal Remodelling, and Endometrial Receptivity

The implantation environment in oocyte-donation IVF markedly varies from that in autologous cycles, since the embryo is completely allogeneic to the recipient. Maternal cells must adapt to the embryo’s whole genetic variation, which introduces a variety of foreign antigens, resulting in a markedly enhanced immunological interface [14]. HLA molecules, including HLA-C, HLA-G, and HLA-E, exhibit both polymorphism and non-polymorphic characteristics on the surface of trophoblasts. Within the decidua, these substances interact with several immunoregulatory systems [15].

The interaction between extravillous trophoblast HLA-C and maternal KIRs, which is mostly located on uNK cells, constitutes a critical molecular barrier. Once considered innate effectors, uNK cells are now recognised as specialised tissue-resident lymphocytes that regulate spiral artery remodelling, release angiogenic factors (VEGF, PlGF), and modify trophoblast invasion [16]. The DAP12 (activating) and DAP10 (inhibitory) adaptor systems regulate downstream activation or inhibition via their interaction with KIR and HLA-C. Inhibitory KIR/HLA-C2 combinations may result in elevated levels of interferon-γ and tumour necrosis factor-α, potentially leading to complications in vascular remodelling, superficial invasion, and implantation failure [17]. In contrast, other activating KIR genotypes, such as KIR2DS1, promote tolerance and regulate trophoblast invasion. In oocyte donation cycles when the foetal HLA-C alleles are exclusively derived from the donor, mismatched KIR-HLA patterns are more probable.

Decidual dendritic cells and macrophages, functioning as antigen-presenting cells, modulate co-stimulatory pathways (CD80/CD86, CD40) and immunological checkpoint molecules (PD-L1/PD-1, CTLA-4) [18]. Dendritic cells may increase the concentrations of PD-L1 and indoleamine 2,3-dioxygenase (IDO1) when exposed to an allogeneic embryo. This would inhibit T-cell proliferation and initiate the catabolism of tryptophan. However, modified dendritic-cell maturation or excessive pro-inflammatory signalling might circumvent tolerance mechanisms [19]. This may inhibit communication between trophoblasts and decidua, hence facilitating the proliferation of Th1-dominant CD4+ T-cells. It is crucial to maintain a balance between pro-inflammatory Th1/Th17 subsets and regulatory Tregs. Post-implantation, Tregs often proliferate as a result of TGF-β and FOXP3 signalling [20]. This renders cells less hazardous to adjacent cells by facilitating their decidualisation. A study on donor egg cycles reveal that some recipients demonstrate reduced Treg recruitment, resulting in a microenvironment that is skewed towards Th1/Th17-type inflammation and insufficient production of TGF-β and IL-10 [21]. A progesterone-primed endometrial matrix undergoing substantial stromal biological remodelling interacts with the allogeneic embryo. DSCs use the cAMP-PKA, FOXO1, GATA2, and NR2F2 pathways to synchronise hormonal signals with immunological responses upon exposure to alloantigens [22]. This results in the production of receptivity mediators such as LIF, IGFBP-1, prostaglandin E2, and integrins including αvβ3. Activation of allostimulus-dependent NF-κB may disturb this balance. This alters the profiles of adhesion molecules that are critical for blastocyst attachment and reduces the amounts of HOXA10 and HOXA11. Transcriptomic investigations of donor egg endometria demonstrate increased activity of chemokines (CXCL9/10/11), pattern-recognition signalling molecules, and complement components (C1q, C3) [23]. Recent single-cell RNA sequencing experiments provide more information: in pregnancies arising from oocyte donation, trophoblast subpopulations have altered the expression of HLA-G isoforms, metalloproteinases (MMP2, MMP9), and angiogenic regulators. Simultaneously, decidual immune compartments exhibit increased numbers of activated uNK subsets and macrophages defined by M1-like transcriptional patterns [24].

### 1.3. Biological Rationale for Glucocorticoid Use in the Peri-Implantation Window

Glucocorticoids have been studied for peri-implantation use due to their direct interaction with the immunological, stromal, and epithelial pathways that govern early embryo–endometrium compatibility. The glucocorticoid receptor alpha (GRα) is a ligand-activated transcription factor that is present in several cell types, including endometrial epithelial cells, decidual stromal cells, uNK cells, macrophages, dendritic cells, and diverse T-cell subpopulations [25]. It is the catalyst for their effects. Upon activation, GRα translocates to the nucleus and modifies gene expression by interacting with transcription factors or binding to glucocorticoid-responsive sites, consequently affecting many implantation-related processes.

One of the primary functions of glucocorticoids is to diminish excessive inflammation. Glucocorticoids impede the production of pro-inflammatory cytokines, including tumour necrosis factor-α, interferon-γ, interleukin-1β, interleukin-6, and interleukin-17, by inhibiting the NF-κB and AP-1 signalling pathways [26]. Inflammatory signals may enhance and disrupt receptivity-related gene networks during oocyte donation cycles, marked by increased alloantigenic stimulation. Glucocorticoids may protect essential molecular factors of implantation, including HOXA10, HOXA11, FOXO1, and integrin αvβ3, which are susceptible to inflammatory suppression, by limiting these pathways [27].

Glucocorticoids influence uterine natural killer cells, which play a crucial role in the remodelling of spiral arteries and are mostly found in the early decidua. Adverse KIR-HLA-C interactions, which are prevalent in donor egg cycles, may render uNK cells more detrimental to other cells [28]. Glucocorticoids diminish cytotoxicity by lowering perforin and granzyme expression, altering interferon-γ production, and fostering a transition towards an interleukin-10 and VEGF secretion profile that enables controlled trophoblast invasion. Glucocorticoids prompt dendritic cells to assume a tolerogenic phenotype in antigen presentation, marked by enhanced synthesis of anti-inflammatory mediators, diminished co-stimulation, and elevated PD-L1 expression [29]. Dendritic cells enhance the proliferation and function of regulatory T-cells by attenuating inflammation induced by Th1 and Th17 cells. This facilitates the maintenance of maternal–foetal tolerance. The glucocorticoid-induced augmentation of this pathway may be especially consequential, given that multiple studies have shown reduced regulatory T-cell recruitment in some donor egg recipients [30].

Decidual cells within the stromal compartment react to glucocorticoids by maintaining the differentiated decidual phenotype and inhibiting dedifferentiation linked to inflammation via the stabilisation of the progesterone-dependent cAMP-PKA-FOXO1 axis [31]. Glucocorticoids alter the functionality of PI3K-AKT and MAPK signalling pathways, resulting in the production of IGFBP-1, prolactin, and prostaglandin E2-crucial mediators that facilitate trophoblast guidance and dietary adaptation. Glucocorticoids also inhibit complement activation. Elevated levels of complement components such as C1q, C3, and C5a are associated with the implantation of donor oocytes. This indicates that the innate immune system is more vigilant. Glucocorticoids mitigate excessive damage to trophoblast cells induced by complement by reducing the expression of complement genes and the recruitment of neutrophils stimulated by C5a. Table 1 delineates the cellular and molecular processes implicated in the implantation of a donor egg and the theoretical influence of glucocorticoids on these pathways.

Table 1 delineates the principal molecular pathways implicated in donor egg implantation. It also indicates how glucocorticoids may influence immunological tolerance, stromal integrity, and trophoblast invasion.

### 1.4. The Evidence Gap in Oocyte-Donation IVF

Despite the distinct immunological properties of oocyte-donation IVF, there is a significant lack of clinical evidence about the use of peri-transfer glucocorticoids in this setting. The bulk of randomised controlled studies and meta-analyses evaluating glucocorticoids in assisted reproduction have been undertaken in autologous IVF populations, where the embryo shares genetic identity with the mother and has a markedly lower alloimmune load [2]. Owing to the unique implantation biology of donor egg recipients, the results of these studies, mainly suggesting no improvement in live birth rates, cannot be accurately generalised to this demographic [32].

Recipients of oocyte donation are often included in small, retrospective studies that exhibit methodological diversity. Glucocorticoids are often used in conjunction with antibiotics, low-dose aspirin, intralipid infusions, granulocyte colony-stimulating factor, or endometrial injury as part of comprehensive “immune-support” treatments [33]. The amalgamation of these therapies obscures the distinct function of glucocorticoids, leaving uncertain whether any enhancement in implantation comes from mechanical signalling, immunomodulation, microbial clearance, or angiogenic effects. The comparability of the research is further hindered by the prevalent lack of defined dosage regimens, scheduling procedures, or immunological phenotype stratification in the majority of publications.

A significant issue is the scarcity of large, well-structured studies targeting immunologically relevant subgroups within the oocyte donor community. Alloimmune profiles, including combinations of activating or inhibitory KIR genotypes, HLA-C mismatch patterns, regulatory T-cell recruitment capability, macrophage polarisation status, and complement activation patterns, demonstrate considerable diversity across donor egg recipients [34]. Clinical trials lack the capacity to identify subgroup-specific advantages without considering these characteristics, thereby neglecting individuals who may experience the most or least benefit from glucocorticoid medication.

Furthermore, the safety statistics are inadequate. Recent findings from extensive recurrent implantation failure (RIF) trials in non-donor IVF indicate apprehensions about heightened biochemical pregnancies, modified placental development, and potential implications for preterm delivery, although the prevailing perception is that short-term glucocorticoid administration is of little risk [35]. The dangers associated with pregnancy with donated eggs remain uncertain in terms of their equivalence, elevation, or reduction compared to natural conception. The unique inflammatory, vascular, and stromal conditions of donor egg implantation may affect glucocorticoid pharmacodynamics; however, this has not been thoroughly assessed.

A further aspect of the evidence gap is that the existing research is unable to use molecular or single-cell profiling to assess therapies. The behaviour of uNK subsets, trophoblast–decidua signalling, dendritic cell tolerogenic programming, and tissue-resident T-cell activation thresholds are all intricately associated with the success of implantation in donor egg cycles [36]. However, no intervention studies have shown a relationship between glucocorticoid administration and molecular biomarkers, including complement-cascade activation, KIR-specific activation signatures, HLA-G isoform expression, or transcriptional trajectories of decidual stromal cells. Table 1 encapsulates the clinical data pertaining to peri-transfer glucocorticoid administration in assisted reproduction, classified by patient demographics, the immunological environment, and result reliability, rather than detailing specific research.

Table 2 presents a conceptual synthesis of clinical information about glucocorticoid medication prior to implantation. The studies are categorised by patient phenotype and immunological environment, rather than by individual research. Glucocorticoids provide no benefits in immunologically unselected populations in assisted reproduction; their advantages are limited to those with certain immune or inflammatory disorders. A significant lack of data in oocyte-donation IVF is the absence of phenotype-stratified randomised studies. Our review primarily discusses information that is derived from mechanistic studies, transcriptome analysis, single-cell profiling, and extrapolation from autologous in vitro fertilisation and recurrent implantation failure populations. The efficacy of peri-transfer glucocorticoid medication in oocyte-donation IVF has not been thoroughly investigated in sufficiently powered randomised controlled studies. Thus, rather than being clinically verified, the immunological and stromal effects described herein should be considered physiologically probable and based on models. The data do not indicate therapeutic effectiveness; nonetheless, they provide a strong theoretical basis for immunomodulation concerning total embryonic allogeneicity. Thus, the therapeutic significance of glucocorticoid use in oocyte-donation IVF remains ambiguous, highlighting the need for rigorously conducted, phenotype-stratified randomised studies with clear safety and effectiveness outcomes.

## 2. Materials and Methods

This narrative review, although not systematic, was developed by utilising a structured and complete methodological approach to guarantee scientific rigour and transparency. An exhaustive and iterative search method was used to identify mechanistic, translational, and clinical information concerning the administration of glucocorticoids prior to implantation in assisted reproduction, specifically targeting oocyte donation cycles. The main databases used were PubMed/MEDLINE, Embase, Web of Science, Scopus, and the Cochrane Library. From the inception of the database until January 2025, searches utilised combinations of terms pertaining to glucocorticoids, embryo implantation, IVF, ICSI, donor oocytes, endometrial receptivity, decidualisation, uterine natural killer cells, autoantibodies, chronic endometritis, reproductive immunology, trophoblast invasion, and implantation failure. To guarantee the thorough identification of relevant material, reference lists from significant studies and current reviews were diligently scrutinised as a component of the search strategy. Only English-language studies were included. This language limitation was implemented to ensure accurate comprehension of scientific, molecular, and clinical data.

Clinical trials or observational studies assessing glucocorticoid administration were during the peri-implantation phase of IVF or donor egg cycles. Mechanistic or translational research investigated the impact of glucocorticoids on endometrial signalling pathways, immune activation, stromal decidualisation, uNK cell function, trophoblast migration, or vascular remodelling. High-quality meta-analyses or narrative reviews addressing the immunological and molecular factors influencing implantation were considered eligible for inclusion. The research on immunogenetics and autoimmunity that clarifies the subgroups that are likely to benefit from glucocorticoid treatment was also included. Research on non-human subjects was often disregarded unless it yielded critical mechanistic insights that were directly relevant to human implantation biology. Case reports, editorials, and conference papers were excluded due to the lack of source data. Studies pertaining to oocyte-donation IVF were included, regardless of the genetic relationship between donor and recipient. Due to inconsistent reporting of donor relatedness in the literature and the genetic distinction of the embryo from the recipient, suggesting an allogeneic immunological environment, instances of family oocyte donation were not thoroughly eliminated.

The selecting procedure of the research occurred in phases. We assessed the relevance of the titles and abstracts obtained from database searches. We obtained the whole texts of all potentially qualifying publications and meticulously examined them to ensure that they aligned with the review’s objectives. Data were extracted narratively, emphasising research design, sample characteristics, glucocorticoid type and dose, time of administration, outcome measures, and possible mechanistic insights. Particular emphasis was placed on comparing outcomes across various clinical groups, including unselected IVF cohorts, patients experiencing recurrent implantation failure, immune-positive individuals, and recipients of donated oocytes. Mechanistic investigations were used to contextualise the clinical results and resolve apparent contradictions in the literature.

The analytical method prioritised conceptual integration, rather than meta-analytic quantification, since the aim of this study was to merge mechanistic knowledge with clinical data. As a result, studies were thematically categorised, based on their contributions to donor-egg-specific physiology, immunology, endometrial biology, or pregnancy outcomes. To provide a comprehensive biological and clinical knowledge of glucocorticoid use in implantation, the intersections and divergences across research were examined. The key objective was to identify inadequacies requiring focused future study and to merge fundamental reproductive immunology with practical clinical decision-making. This study only examines oocyte-donation IVF cycles, when the recipient of the eggs gestates the pregnancy herself. Models of gestational surrogacy were omitted because of their unique biological and immunological contexts.

## 3. The Immunological Landscape of Oocyte-Donation IVF

Giving away oocytes for IVF creates one of the most unique immunological environments for human reproduction. The fully allogeneic embryo, which contains genetic material that is completely foreign to the recipient, interacts with the mother’s immune, stromal, and vascular systems via molecular pathways that are fundamentally distinct from autologous cycles. This section summarises what we currently know about the immune cell populations, signalling pathways, and tissue-level interactions that happen during implantation in people who receive donor eggs.

### 3.1. Tissue-Resident Immune Compartments and Alloimmune Activation

During early pregnancy, the decidua has a sophisticated immunological environment that facilitates embryo implantation while maintaining maternal immune health. In oocyte-donation IVF, this system encounters a wholly allogeneic embryo, resulting in a significantly altered immunological milieu compared to autologous cycles. The increased antigenic disparity between trophoblast and maternal tissues results in elevated activation thresholds, alterations in cellular phenotypes, and enhanced molecular signalling across many immune cell compartments.

### 3.2. Primary Regulators of Elevated Alloimmune uNK Cells

During the implantation window, about 70% of immune cells are NK cells, the predominant form of leukocyte in the early decidua [37]. uNK cells are tissue-resident, non-cytotoxic lymphocytes that primarily regulate angiogenesis, vascular remodelling, and trophoblast invasion. This differs from peripheral NK cells. Maternal KIR (killer immunoglobulin-like) receptors and foetal HLA-C ligands on extravillous trophoblasts interact to modulate their activity [38].

The embryo has a unique HLA-C genotype, significantly increasing the likelihood of KIR/HLA-C incompatibility during oocyte donation cycles. This discrepancy may result in increased uNK reactivity via inhibitory KIR2DL1-HLA-C2 or activating KIR2DS1-HLA-C2 pairings [39]. Inhibitory KIR interaction particularly amplifies TNF-α release and limits EVT (extravillous trophoblast) infiltration, while fostering an IFN-γ-predominant NK profile. Single-cell RNA sequencing studies indicate that donor egg implantation locations include a greater abundance of “activated” uNK cell subsets. These subgroups are characterised by elevated levels of CXCL10, IFNG, PRF1, and GZMB, and heightened expression of angiostatic chemokines. These modifications may jeopardise the typical remodelling of spiral arteries by shifting the balance from regulatory to inflammatory phenotypes [40].

Decidual macrophages often oscillate between M1-like (pro-inflammatory) and M2-like (tolerogenic) states to facilitate tissue remodelling, angiogenesis, and the clearance of apoptotic cells [41]. Transcriptomic investigations reveal increased M1/M2 heterogeneity in donor egg cycles, with certain subgroups demonstrating enhanced expression of TNF-α, CD86, IL-1β, and nitric oxide synthase pathways. This biassed polarisation diminishes the synthesis of TGF-β and IL-10, complicating implantation due to an unfavourable regulatory environment. Malfunctioning macrophages may inhibit the remodelling of the extracellular matrix and impede the mobility of trophoblasts. This is due to their regulation of the matrix composition via the production of MMPs and TIMPs [42].

Dendritic cells, crucial antigen-presenting cells in the decidua, regulate T-cell activation and tolerance. During donor egg implantation, dendritic cells exhibit elevated amounts of MHC class II, costimulatory molecules (CD80/CD86), and components of the antigen-processing machinery [43]. This characteristic indicates that the foreign embryo is more effective in seeking alloantigens. However, when dendritic cells undergo excessive maturation, their propensity to differentiate into tolerogenic DCs diminishes. Tolerogenic DCs are often responsible for producing IDO1, PD-L1, and retinoic acid-dependent immunoregulatory signals, as well as promoting the proliferation of regulatory T-cells. When dendritic cells fail to adopt a tolerogenic pattern, immunological tolerance is compromised, leading to an enhanced uterine response to the Th1 signals [44].

Adaptive T-cell modulation significantly influences maternal tolerance. Typically, Tregs (FOXP3+) significantly increase in size following implantation. This inhibits the activation of cytotoxic T-cells and induces the production of TGF-β and IL-10. In donor egg cycles, there is less Treg localisation, increased Th1 cytokines such as IFN-γ and IL-2, and elevated levels of IL-17, which is associated with Th17 [45]. The disparity between Treg and Th1/Th17 subsets exacerbates the inflammatory nature of the alloimmune response. This may result in superficial invasion, complement activation, and inadequate decidualisation.

C1q, C3, and C5a are complement proteins with distinct roles during implantation. Excessive complement activation, often seen in donor egg endometria, may result in increased neutrophil recruitment, collateral tissue damage, and enhanced inflammatory cytokine release [46]. C1q promotes trophoblast adhesion. Single-cell transcriptomics of donor egg implantation locations reveal elevated levels of C3, CFB, CFD, and C5aR1. This indicates that the innate immune system is more vigilant, perhaps restricting trophoblast proliferation and complicating vascular remodelling [47].

### 3.3. Cytokine Gradients, Chemokine Networks, and Molecular Crosstalk

Cytokine and chemokine signals intricately collaborate to regulate the implantation environment and instruct immune, stromal, epithelial, and trophoblast cells on their functions. The embryo in oocyte-donation IVF has an antigenic profile that is entirely distinct from that of the mother, making this network more dynamic and susceptible to dysregulation. The enhancement, alteration, or extension of cytokine gradients and chemokine pathways that usually govern receptive endometrial changes creates an immune environment that is different from that seen in autologous cycles.

Leukaemia inhibitory factor (LIF), granulocyte macrophage colony stimulating factor, IL-6, and IL-8 are the primary molecules regulating the transient inflammatory response of the endometrium during a typical implantation window. These cytokines initiate phosphorylation cascades via the JAK–STAT3, ERK1/2, and PI3K-AKT pathways [48]. This facilitates stromal decidualisation, epithelial rearrangement, and the expression of critical receptivity markers. LIF-mediated STAT3 activation enhances the transcription of genes that are essential for implantation, including EGR1 and SOCS3. This enhances epithelial adherence and integrin aggregation. IL-8 facilitates initial angiogenic sprouting and neutrophil-driven extracellular matrix remodelling, whereas IL-6 operates at the stromal level to sustain progesterone-dependent differentiation [49].

Nonetheless, heightened awareness of foetal alloantigens following donor egg implantation often exacerbates the first inflammatory response. Increased activity of pattern-recognition receptors such as TLR2, TLR4, and the NLRP3 inflammasome leads to elevated production of IL-1β, TNF-α, IFN-γ, and IL-17. These cytokines disrupt progesterone-mediated changes in epithelial and stromal cells by influencing the NF-κB, STAT1, and IRF signalling pathways [50]. They also inhibit the transcription of receptivity determinants such as HOXA10, HOXA11, and integrin αvβ3. Transcriptomic analyses of donor egg implantation sites indicate a shift from a receptive, STAT3-dominant milieu to an inflammatory, STAT1-driven condition, marked by the enrichment of NF-κB target genes, the increased predominance of STAT1/STAT4 over STAT3, and the upregulation of interferon-stimulated genes [51].

The cytokine polarisation is seen in the chemokine landscape. Increased interferon signalling correlates with a greater likelihood of transcription for CXCL9, CXCL10, and CXCL11. These are very effective in attracting uNK and effector T-cells that express CXCR3 on their surface [52]. Chemotactic gradients extend Th1-biassed inflammation and promote leukocyte recruitment. Simultaneously, elevated concentrations of CCL2 and CCL5 recruit T-cells and monocytes to the decidua. This induces the proliferation of inflammatory macrophage subsets in a localised region and enhances the potency of cytokine amplification loops. Conversely, signals such as CXCL12-CXCR4/CXCR7 interactions, which typically regulate trophoblast migration, begin to malfunction. Moreover, the diminished integrity of the CXCL12 gradients may contribute to the shallower invasion often seen in donor egg implantation deficiencies, in addition to disrupting regulated EVT migration [53].

Alterations in cytokines and chemokines modify the communication between trophoblasts and the decidua. Extravaginal trophoblasts often engage with maternal LILRB1, KIR2DL4, and NK2G receptors to facilitate immunological tolerance via the expression of HLA-G, HLA-E, and non-classical HLA-F [37]. In pregnancies using donor eggs, increased concentrations of antagonistic cytokines, such as IFN-γ and TNF-α, affect EVT signalling by modulating the expression of HLA-G isoforms and altering trophoblast transcriptional programmes to bolster cellular defence systems [54]. These modifications influence downstream invasion regulators, including MMP2, MMP9, TIMP1, and TIMP3, hence disrupting the proteolytic equilibrium that is essential for extracellular matrix remodelling. Decidual stromal cells are susceptible to dedifferentiation induced by cytokines when STAT1 and NF-κB are persistently activated. The cAMP-PKA-FOXO1 pathway is essential for the maintenance of cellular differentiation [55]. Secretion of IGFBP-1 and prolactin is impaired, NRF2-mediated mitochondrial oxidative-responsive signalling is reduced, and many metabolic pathways that are essential for early trophoblast feeding are disturbed.

In this cytokine-rich milieu, the integration of signals is crucial for successful implantation. When STAT3 is deactivated, the transcriptional mechanisms that render the endometrium responsive are diminished in efficacy [56]. However, when STAT1 and NF-κB interact, they establish a collaborative inflammatory circuit. Increased MAPK and JNK activity disrupts the cytoskeletal dynamics that are necessary for blastocyst adhesion and fosters instability in epithelial tight junctions. Malfunctioning chemokine receptors may lead to excessive activation of PI3K-AKT, resulting in diminished decidual resilience by altering glucose utilisation and impairing autophagic responses during trophoblast invasion [57].

### 3.4. Functional Implications for Implantation

The functional behaviour of the implantation site epitomises the completion of the immunological and molecular processes that define oocyte-donation IVF. Successful implantation requires the precise coordination of controlled inflammation, trophoblast invasion, stromal decidual stability, vascular remodelling, and epithelial receptivity [1]. The coordination becomes much more difficult in the situation of a wholly allogeneic embryo. The elevated levels of cytokines, chemokines, and cellular signals that were previously discussed provide an implantation milieu that must facilitate enhanced alloantigen recognition while also maintaining a rigorously regulated tolerance programme. Alterations in this equilibrium, whether vascular, stromal, or inflammatory, result in functional impairments throughout the first phases of placental development [58].

One of the first functional impacts is the alteration of trophoblast invasion depth, which is highly responsive to inflammatory signals. Increased concentrations of IFN-γ, TNF-α, and IL-17 impede trophoblast motility by interfering with STAT3-dependent transcription and inducing the production of anti-invasive proteins such as TIMP1 and TIMP3 [59]. These cytokines modify trophoblast intracellular signalling by enhancing the activities of STAT1 and IRF1. This results in defensive, rather than pro-invasive, traits. Consequently, extravillous trophoblasts may have altered cytoskeletal dynamics, and a decreased ability to invade the decidua and remodel the spiral arteries, as well as lower activity of MMP2 and MMP9. This results in subclinical implantation failure, placental insufficiency, and shallow implantation, which are prevalent functional issues that may lead to early pregnancy loss [60].

Simultaneously, persistent inflammatory signalling renders the decidual stromal compartment unstable. Stromal cells depend on the cAMP-PKA-FOXO1 pathway, progesterone receptor function, and the equilibrium of PI3K-AKT and MAPK signalling to maintain their differentiated phenotype [61]. Excessive activation of NF-κB and STAT1 disrupts this equilibrium, resulting in diminished FOXO1 transcriptional activity and impeding the synthesis of prolactin and IGFBP-1, which are both critical indicators of full decidualisation. Stromal dedifferentiation reduces the decidua’s ability to support trophoblast anchoring and jeopardises matrix organisation [62]. During the metabolically intensive implantation period, inhibiting NRF2-mediated inflammation renders cells more vulnerable to oxidative stress by diminishing their defensive capabilities. These deficits impair the structural, metabolic, and immunological activities of the decidua.

Vascular transformation is a critical functional milestone that is at risk. Optimal remodelling of the maternal spiral arteries necessitates collaboration between uNK cells and invasive trophoblasts [62]. However, when uNK cells exhibit increased cytotoxicity or pro-inflammatory characteristics, they secrete reduced levels of VEGF-A, IL-10, and angiogenic growth factors. This occurs due to suboptimal KIR-HLA-C interactions and increased IFN-γ signalling. Concurrently, enhanced CXCL9/CXCL10 signalling alters the activation state and spatial distribution of uNK subsets, hence complicating their ability to regulate endothelium remodelling and the apoptosis of vascular smooth muscle cells. Incomplete arterial transformation is marked by a constricted luminal diameter, intact vascular smooth muscle, and insufficient blood supply to the growing implantation site [63].

Excessive cytokines may impair the adhesion processes associated with epithelial receptivity. Elevated MAPK/JNK signalling, diminished integrin αvβ3 expression, and decreased LIF and STAT3 activity disrupt glycocalyx remodelling and epithelial polarity. These alterations establish the first functional impediments to implantation by complicating the attachment and cohesion of the blastocyst, even prior to trophoblast invasion [64]. The immune system is hyperactive, imposing increased metabolic and energy strain on the implantation microenvironment. Metabolic pathways induced by interferon augment the glycolytic flow and the generation of reactive oxygen species in epithelial and stromal cells. If these alterations are not counterbalanced by antioxidant responses, they may damage the mitochondria and diminish the ATP required for decidual growth, extracellular matrix remodelling, and trophoblast-mediated invasion [65].

## 4. Clinical Evidence for Peri-Transfer Glucocorticoids in IVF

The extensive but fragmented clinical literature on glucocorticoid treatment in assisted reproduction is difficult to interpret because of significant methodological issues, variations in patient selection, and a notable lack of data about oocyte-donation cycles. Despite the use of glucocorticoids in IVF for over thirty years, their efficacy in practice, particularly regarding live birth outcomes, remains ambiguous [25]. To comprehensively evaluate the advantages and disadvantages of glucocorticoid treatment, it is essential to examine studies in three primary domains: autologous IVF, RIF, and oocyte-donation IVF.

### 4.1. Evidence from Autologous IVF Trials and Meta-Analyses

More than thirty years of clinical research on peri-transfer glucocorticoids in autologous IVF have produced astonishingly similar findings: glucocorticoids have not shown significant enhancements in live birth rates, despite substantial biological plausibility [66]. The majority of the evidence originates from randomised studies and meta-analyses investigating the potential of glucocorticoids to enhance embryo implantation via immune suppression, inflammation reduction, and endometrial stabilisation.

During the 1990s and early 2000s, the first randomised studies used prednisone, prednisolone, methylprednisolone, or dexamethasone. The dose regimens varied from brief courses given just before transfer to extended ones that started during ovarian stimulation [2]. These studies often included different IVF populations with various therapeutic reasons, such as tubal disease, male-factor infertility, and idiopathic infertility. The majority of patients probably lacked any underlying immunological disease, since few investigations included biological markers that were suggestive of inflammation or immune activation [67].

Recent meta-analyses, including these preliminary studies, indicate that glucocorticoid therapy does not enhance the likelihood of achieving a live delivery. A restricted amount of research indicated slight enhancements in clinical pregnancy rates [68]. Nevertheless, these findings often stemmed from small sample numbers, showed variability, and lacked statistical rigour. Subsequent re-analyses indicated that trials with methodological flaws, inadequate blinding, varying embryo quality, or fluctuating luteal support often explained the observed advantages. Furthermore, blastocyst culture, vitrification, trophectoderm biopsy, and time-lapse selection, each significantly affecting implantation rates and reducing the probability of detecting minor treatment effects, were mostly missing from previous experiments [69].

As IVF techniques progressed, new randomised studies sought to assess glucocorticoids in standardised laboratory settings. Nevertheless, these latest trials failed to show any improvement in live birth rates, continued pregnancies, or implantation, indicating that the negative results cannot be solely attributed to an obsolete methodology [35].

A multitude of mechanistic explanations has been proposed to explain why this remains ineffective. The endometrium depends on a natural inflammatory surge during implantation, mediated by uNK activation, LIF-STAT3 signalling, IL-6, and IL-8 [70]. Glucocorticoids may unintentionally obstruct pathways that are critical for early trophoblast invasion and embryo attachment by reducing this inflammatory response. Secondly, autologous IVF does not create a markedly alloantigenic immunological environment. The partial genetic similarity between the mother and embryo reduces the activation of pathways that glucocorticoids usually regulate, such as KIR/HLA-C-mediated uNK responses. This likely diminishes the efficacy of immunosuppressive therapy during standard IVF cycles [71].

A significant influence is the variation in glucocorticoid exposure. Treatment duration may vary from three days to three weeks, with dosages ranging from “physiological replacement” (5 mg prednisone per day) to somewhat pharmacological (20–30 mg per day) [72]. These discrepancies complicate the determination of whether adverse results result from inadequate medication dosage or whether the medication is inherently ineffective. Timing is critical: many trials started glucocorticoids either too late, after the limited phase of endometrial immune changes, or too early, during controlled ovarian stimulation, when endometrial effects are negligible.

Recent meta-analyses from the last decade to fifteen years indicate that the few positive studies are often outliers and have a significant risk of bias. Furthermore, even when concentrating on certain subgroups, such as women with positive autoantibodies, no meta-analysis has shown a statistically significant increase in live birth rates. Together, the results provide compelling evidence against the use of glucocorticoids in unselected autologous IVF cohorts [73]. This contrasts significantly with oocyte-donation IVF, which lacks robust clinical proof but has a more compelling biological rationale due to complete foetal allogeneicity.

### 4.2. Recurrent Implantation Failure and Immunologically High-Risk Groups

Immunomodulatory therapies, such as glucocorticoids, have traditionally been considered the most effective in instances of RIF. We believe this is due to the possibility that some women experiencing recurrent IVF failures may have issues related to endometrial receptivity, cytokine signalling, immunological activation, or maternal–foetal tolerance [35]. Historically, the phrase “immune-mediated RIF” included a range of conditions, including elevated peripheral NK-cell activity, abnormal cytokine profiles, the existence of autoantibodies, and unexplained implantation failure [74]. Numerous early investigations used varying thresholds to delineate “immune abnormalities,” utilised non-standardised immunological procedures, and lacked clear diagnostic criteria [75].

Multiple small cohorts and non-randomised trials from previous clinical research suggest that glucocorticoids, often used with antibiotics, low-dose aspirin, heparin, or intralipid treatment, may enhance pregnancy rates or implantation success in individuals with RIF [35]. Retrospective analyses often demonstrated improved clinical pregnancy rates after treatment with methylprednisolone or prednisolone, especially in women showing presumed indicators of immune activation, such as increased CD56+ uNK cells or positive ANA/anticardiolipin antibodies [76].

The commencement of rigorous randomised controlled studies aimed at immunologically high-risk women marked significant progress in this field. The primary study is a substantial, multi-centre, placebo-controlled randomised controlled trial examining low-dose prednisone in well-defined recurrent implantation failure patients [77]. This experiment included stringent criteria for participant eligibility, uniform definitions of RIF in biopsies, and dependable methods for outcome measurement. The conclusive findings demonstrated that prednisone did not improve implantation, clinical pregnancy, or live birth rates. The research identified possible harmful effects, including increased rates of biochemical pregnancy loss. These data challenge the notion that immune suppression is advantageous for RIF patients and indicate that severe suppression of physiological inflammatory pathways, especially uNK-mediated angiogenic signalling and IL-6/LIF/STAT3, may obstruct proper implantation [35,78].

The lack of effect in the RCT raises substantial mechanistic issues. Regulated inflammatory signalling is integral to several immune system functions that are critical for implantation, including the activation of NF-κB, transient release of Th1 cytokines, modulation of epithelial cells from IL-6 to STAT3, and targeted activation of uNK cells [79]. In the absence of pathological inflammation, glucocorticoids may disrupt these processes by generally suppressing the expression of NF-κB and AP-1 regulated genes [35].

Another complex aspect is that peripheral blood NK cell testing continues to be used, despite the absence of data linking it to uterine NK cell activity. Previously, the term “high NK activity,” now deemed outdated, was used to justify different RIF treatments. Cytokine panels assessing peripheral Th1/Th2 ratios do not accurately reflect the tissue-level immunological milieu of the decidua [80]. The improper use of glucocorticoids in RIF groups without immunological dysregulation was mainly due to inconsistency between diagnostic tests and pertinent immune biology. Women with chronic endometritis, defined by ongoing stromal plasma-cell infiltration, elevated IL-1β and TNF-α production, and altered epithelial regeneration, are a particularly important subpopulation who are often investigated in RIF investigations. Chronic endometritis, if misdiagnosed or untreated, may both mimic and obscure any possible glucocorticoid impact. It responds to antibiotics, rather than steroids. Upon reflection, several publications regarding the advantages of glucocorticoids in RIF may mostly pertain to the concealed effects of antibiotics or steroids on the immune system, rather than alterations to the immune system itself [81].

### 4.3. Clinical Evidence in Oocyte-Donation IVF

The clinical data examining glucocorticoid medication in donor egg recipients is very few, methodologically varied, and insufficient for making decisive judgements, despite the strong biological justification for immunomodulation in oocyte donation IVF [73]. Donor egg cycles exemplify the most immunologically unique model in assisted reproduction, marked by total foetal allogeneicity, enhanced KIR/HLA-C-mediated uNK activation, elevated STAT1-dominant cytokine signalling, and an increased reliance on tolerogenic dendritic cells and Treg networks [82].

The lack of data arises from the design of most previous glucocorticoid studies, which either excluded donor egg recipients or included them in inadequate numbers without independent analysis. Owing to the significant immunological differences between the two systems, practically all conclusions about the effectiveness of glucocorticoids in donor egg IVF have been based on autologous or recurrent implantation failure research, which represents a medically faulty approach [35,83]. During donor egg implantation, trophoblasts display non-self HLA-C ligands, decidual dendritic cells show enhanced antigen presentation, and maternal uNK cells have increased inhibitory KIR–HLA mismatches [84]. These allogeneic contacts trigger inflammatory pathways that are either non-existent or significantly reduced in autologous IVF, including complement activation, CXCL9/10/11 chemotactic axis, interferon-stimulated genes, and inflammatory macrophage polarisation. In glucocorticoid clinical studies, none of these immunological reactions have been thoroughly assessed.

The current clinical evidence on donor egg cycles mostly comprises retrospective cohorts, small observational studies, and uncontrolled series; several research used glucocorticoids in multimodal “immune-support” procedures [35]. Prednisone or methylprednisolone was often provided in conjunction with therapies such as G-CSF, intralipid emulsions, heparin, low-dose aspirin, or doxycycline [85]. For instance, rather than genuine immunosuppression, some “steroid-responsive” enhancements may really result from the treatment of chronic endometritis, alterations in subclinical bacterial biofilms, or enhanced blood flow induced by G-CSF.

Limited sample numbers and insufficient methodological rigour defined the few donor egg investigations that directly evaluated glucocorticoids. A restricted set of outcome measures assessed live birth, whereas others focused on biochemical pregnancy, clinical pregnancy, or implantation rates [86]. Significantly, none of the investigations included immunological phenotyping, suggesting that factors such as patients’ immunogenetic history, cytokine milieu, uNK phenotype, stromal transcriptome profile, or complement activation status were overlooked during glucocorticoid treatment. A foetal HLA-C2 embryo, paired with the mother’s KIR AA genotype, may result in a significantly intensified uNK-mediated inflammatory response, whereas KIR B haplotypes may provide a notably more tolerant environment [39]. Donor egg studies lack the requisite precision to identify subgroup-specific advantages that may be concealed when all recipients are examined collectively without stratification.

A significant issue with the existing research is its insufficient emphasis on the timing of therapy, which is crucial in donor egg cycles. The increase in T regulatory cells and tolerogenic dendritic cells, changes in decidual NF-κB activity, and the activation of STAT3-mediated receptivity pathways define the critical immunological changes that determine implantation success within a limited time period [87]. Glucocorticoids given outside of these intervals may not reduce pathological inflammation but instead obstruct physiological remodelling. However, the majority of donor egg investigations use steroids experimentally, without any mechanistic explanation, from the commencement of progesterone priming to the assessment of β-hCG [2].

Furthermore, donor egg cycles lack safety data. The distinctive immunological milieu of donor egg placentation raises theoretical concerns about the possible excessive suppression of pathways that are critical for trophoblast invasion, arterial remodelling, and the early phases of placental vascular development, notwithstanding the prevailing agreement that short-course glucocorticoids are safe during pregnancy [84]. The growing data from comprehensive RIF studies suggests that glucocorticoids may affect placental development and increase biochemical pregnancy loss, highlighting the need for safety assessments related to donor eggs. The risks associated with hypertensive diseases, preeclampsia, and aberrant placentation are heightened in donor pregnancies and may be influenced by trophoblast invasion or inflammation. The impact of glucocorticoids on these hazards remains wholly ambiguous [25,35].

## 5. Bridging Mechanistic Theory with Clinical Reality

Although clinical data supporting the efficacy of glucocorticoids is limited, a compelling biological rationale for their use in oocyte donation IVF exists. To comprehend this distinction, an integrated examination of how molecular pathways contribute to, or fail to contribute to, clinically significant enhancements in implantation, placentation, and pregnancy outcomes is required. Clinical investigations have not yet integrated these mechanistic insights into research design, patient selection, scheduling, or endpoint assessment, despite the fact that donor egg implantation is affected by distinct immunogenetic interactions, vigorous cytokine signalling, and specialised stromal and vascular dynamics. This section explains why, despite strong theoretical underpinnings, glucocorticoids have failed to show consistent clinical success by synthesising the molecular biology of donor egg implantation with the current clinical evidence.

### 5.1. Why Biological Plausibility Does Not Ensure Clinical Efficacy

Biological plausibility alone may not ensure enhanced clinical results, despite the strong molecular justification for glucocorticoid treatment in oocyte donation IVF. Implantation, a meticulously orchestrated biological process, requires the simultaneous occurrence of inflammation, immunological tolerance, stromal differentiation, angiogenesis, extracellular matrix remodelling, and trophoblast invasion at a specific locale [88]. The broad effects of glucocorticoids, which seem beneficial from a molecular perspective, may paradoxically compromise their clinical efficacy.

The dual function of inflammation during implantation presents a considerable problem. While excessive inflammation is detrimental, a regulated inflammatory response is essential to initiate the critical receptivity pathways. The receptive endometrium experiences a transient, precisely controlled surge of IL-6/LIF/STAT3, TNF-α, IL-8, and NF-κB signalling to promote angiogenic priming, stromal activation, and epithelial remodelling [89]. Glucocorticoids may inadvertently reduce these physiological signals via GRα-mediated transrepression of NF-κB and AP-1. For example, decreasing NF-κB-induced CXCL8 diminishes neutrophil-mediated extracellular matrix degradation, whilst inhibiting STAT3 shifts the balance towards STAT1-dominant signalling, undermining the transcriptional programmes that are necessary for adhesion and initial trophoblast contact [90]. Thus, the therapeutic window for glucocorticoids to alleviate pathological inflammation while preserving physiological inflammation is very limited, even in donor egg cycles marked by increased inflammation.

The significant redundancy and adaptability of endometrial immune networks constitute the second issue. The regulation of cytokine and chemokine signalling occurs via concurrent mechanisms. When one axis is deactivated, other axes are often activated to compensate [91]. Glucocorticoid-induced inhibition of TNF-α and IL-1β may unintentionally amplify interferon-dependent IRF1/STAT1 pathways by interfering with negative crosstalk. Similarly, reducing the activation of dendritic cells may diminish the efficacy of tolerance mechanisms by decreasing the presentation of local antigens and impairing the induction of IDO1 and PD-L1. Consequently, broad anti-inflammatory agents, such as glucocorticoids, may not improve the immunological milieu and might even exacerbate existing abnormalities [92].

A significant issue with clinical translation is achieving optimal timing. The sequence of progesterone priming, epithelial differentiation, uNK cell activation, and EVT engagement constitutes a restricted immunological timeline that governs implantation [93]. Despite the critical immune-modulatory window lasting just a few days, most clinical protocols provide glucocorticoids for an extended duration, often from the initiation of progesterone treatment until the β-hCG assessment. If glucocorticoids are administered too late, critical processes like uNK activation, Treg recruitment, and stromal FOXL2/FOXO1-mediated differentiation will have already occurred [94]. If administered prematurely, the endometrium may not exhibit the inflammatory activation that glucocorticoids are intended to regulate. If glucocorticoid treatment is not precisely synchronised with biological processes, it may fail to reach its mechanistic aim. No clinical study has undertaken this yet [35].

The variability across patients, even within the donor egg group, is a significant drawback. Significant biological plausibility is shown in women with increased alloimmune activation, particularly those possessing a maternal KIR AA genotype with HLA-C2 blastocysts, which are noted for their reduced angiogenic ability and elevated IFN-γ production in uNK cells [95]. Nevertheless, clinical research has used glucocorticoids indiscriminately, without classifications based on immunogenetic profiles, cytokine signatures, uNK transcriptomes, stromal inflammatory markers, or complement activation [96].

The intricacy of trophoblast-decidual communication poses a further hurdle. Glucocorticoids influence both maternal and embryonic compartments. Inhibiting decidual inflammation may improve tolerance, but it may also reduce trophoblast invasion, spiral artery remodelling, and autocrine survival signals [97]. Excessive exposure to glucocorticoids may alter VEGF-A-mediated angiogenesis, disrupt the MMP2/MMP9 proteolytic equilibrium, or diminish the amounts of HLA-G isoforms on EVT cells. Suppressing detrimental inflammation while permitting trophoblast invasion is challenging. This is likely unfeasible with systemic steroids that lack spatial specificity [98]. These biological concerns are amplified by methodological shortcomings in clinical studies. Many studies use endpoints that inadequately reflect the efficacy of implantation, such as biochemical or early clinical pregnancy outcomes. Inflammation affects deep decidual invasion, vascular remodelling, placentation, and the apposition and attachment of the blastocyst. Comprehensive RIF studies have shown an increase in biochemical pregnancy rates without a parallel improvement in continuing pregnancy rates. This suggests that glucocorticoid inhibition of initial inflammatory responses may temporarily enhance biochemical implantation while concurrently compromising deeper implantation processes [35].

### 5.2. Potentially Responsive Subgroups in Oocyte-Donation Cycles

Certain physiologically plausible subgroups within the oocyte-donation framework may exhibit differing, perhaps increased, responses to specific peri-implantation immunomodulation, even if the general data does not support the regular use of glucocorticoids in unselected IVF populations [99]. These subgroups arise from extensive immunogenetic, cellular, transcriptomic, and decidual-stroma-specific investigations that uncover unique patterns of alloimmune activation [100].

Women exhibiting molecular markers of stromal inflammatory dysregulation, such as increased NF-κB activity, decreased FOXO1 and IGFBP-1 expression, impaired progesterone-receptor signalling, and weakened decidualisation signatures, are another potentially responsive category [101]. These anomalies lead to a “pre-inflammatory decidua” that is vulnerable to NF-κB-mediated EVT suppression and acutely responsive to trophoblast contact. Glucocorticoids may theoretically rectify this imbalance by re-establishing the balance of PI3K AKT-dependent metabolic pathways that are essential for stromal resilience, halting the inhibition of matrix metalloproteinases induced by prostaglandins and reinstating the transcriptional integrity of FOXL2/FOXO1. Steroids may not only reduce inflammation in these women but may also restore compromised decidual cellular activities that are critical for implantation [35].

Numerous donor egg transcriptome investigations have shown that a subset has elevated levels of interferon-stimulated genes (ISGs), including IFIT1/3, OAS1/2, CXCL9/10/11, and IRF7. ISG-high endometria often display increased Th1 skewing and show hyperreactivity to trophoblast interactions [102]. Glucocorticoids may be especially efficacious in reducing this ISG signature and re-establishing equilibrium between Th1-dominant activation and implantation-permissive tolerance, since they reduce STAT1 phosphorylation and mitigate interferon-mediated gene expression [103].

Dysregulation of complement, marked by increased expression of decidual C3, C5aR1, or CFB, may result in collateral tissue injury, enhanced neutrophil infiltration, and compromised trophoblast anchoring [104]. Similarly, certain receivers of donor eggs exhibit significant polarisation of M1 macrophages, marked by the upregulation of TNF-α, IL-1β, and nitric oxide synthase pathways. Glucocorticoids promote macrophage polarisation towards anti-inflammatory M2 phenotypes, inhibit complement transcription, and reduce C5a-mediated chemotaxis. Patients demonstrating implantation failures due to inflammation linked to complement or macrophages may represent a unique responder category [105].

Chronic endometritis (CE), characterised by micro-inflammatory damage, plasma-cell infiltration, increased IL-1β/TNF-α expression, and compromised decidualisation, is more prevalent than anticipated among receivers of donor eggs [106]. While CE is fundamentally an infectious condition requiring antibiotics, glucocorticoids may provide further advantages for individuals displaying “post-inflammatory stromal rigidity,” marked by prolonged inflammatory gene expression after microbial eradication. Steroids should not be the exclusive therapy for chronic eczema. Any potential advantage comes from re-establishing stromal equilibrium post-infection, rather than from mitigating inflammation induced by the infection. This is speculative and deserves more evaluation [107].

Emerging single-cell data identify donor egg recipients whose uNK subsets have increased cytotoxic transcripts (e.g., PRF1, GZMB), heightened IFNG levels, or reduced angiogenic mediators. The hyperreactive uterine natural killer phenotype in these individuals obstructs extravillous trophoblast invasion [11]. Glucocorticoids promote NKG2A-dominant states characterised by tolerance, reduce the levels of GZMB and PRF1, and alter the activation thresholds of uNK cells. In this subgroup, the tailored use of glucocorticoids is physiologically rational and may provide benefits [108]. Dysregulation of the receptive window, characterised by abnormal endometrial “receptivity array” signals and heightened inflammation, is seen in a fraction of receivers of donor eggs. If dysregulation originates from the immune system, rather than hormonal factors, glucocorticoids may assist in aligning immunological signals with the implantation window, promoting synchronisation [35].

A common theme among these subgroups is that glucocorticoids are improbable to benefit the average donor egg recipient. However, they may be beneficial for women whose implantation failure is linked to particular, identifiable immune abnormalities, such as STAT1-dominant inflammatory signalling, KIR/HLA-C-driven uNK hyperactivation, interferon-enriched endometrial transcriptional profiles, or excessive complement activation [109]. The main challenge in finding these responder phenotypes is the lack of biomarker-stratified studies. Thus, immunogenetic characterisation, including KIR/HLA-C compatibility, must be included in future randomised trials with cytokine and chemokine profiling, uNK-cell transcriptome signatures, complement activity indicators, and decidualisation competency indices. Glucocorticoids may be assessed only within certain biological situations where their methods of action correspond with the underlying pathophysiology of implantation via carefully structured investigations [110]. Table 3 enumerates the immunological phenotypes for which peri-transfer glucocorticoids may confer advantages in oocyte-donation IVF, grounded in contemporary mechanistic and clinical evidence.

Table 3 shows possible immunological subgroups of people who might benefit from targeted peri-implantation glucocorticoid therapy after obtaining oocyte donations. The necessity for biomarker-guided randomised trials is underscored by the current evidence being indirect and inadequate for standard clinical use.

### 5.3. Safety Considerations in Donor Egg Pregnancies

Peri-implantation glucocorticoid medication is often considered to be safe in the first phases of pregnancy. This assumption is mostly based on spontaneous conception and autologous IVF, rather than oocyte donation cycles [2]. The distinctive immunological structure of donor egg pregnancies is characterised by complete foetal allogeneicity, enhanced uNK trophoblast contacts, and a heightened baseline risk of placentation diseases. The use of systemic glucocorticoids during the crucial peri-implantation period raises safety concerns due to these features.

Consideration of the impact of glucocorticoids on trophoblast invasion and the first phases of placental development is essential. A precise equilibrium between immunological tolerance and regulated inflammation is essential for appropriate EVT development [111]. Excessive suppression of NF-κB and STAT3 signalling may reduce the IL-6/LIF-mediated differentiation signals that promote trophoblast adhesion, motility, and spiral artery remodelling. Obstructing these routes may result in superficial implantation, inadequate vascular transformation, or alterations in placental perfusion. These hazards are already more prevalent in pregnancies using donor eggs, which are associated with increased incidence of placental malfunction, preeclampsia, and hypertensive diseases [112].

Glucocorticoids modify the biology of uNK cells in manners that may not consistently favour donor egg situations. Overly suppressing angiogenic uNK subsets may diminish the production of VEGF-A and PlGF, which are essential for vascular growth and initial placental haemodynamics, whereas moderating uNK hyperactivation may safeguard against excessive IFN-γ-induced trophoblast inhibition [113]. An excessively strong glucocorticoid impact may inadvertently disrupt these compensating systems. Donor egg placentation is characterised by heightened uNK activation and a higher need for angiogenic support. The impact on metabolism and epigenetics is another aspect of safety [114]. Glucocorticoids may influence oxidative-stress pathways, intensify insulin resistance, and alter maternal glucose utilisation via influencing gene transcription through GRα. Imbalances in oxidative equilibrium may impact cellular function in the early placental environment, where trophoblasts rely on meticulously controlled ROS signals for invasion and mitochondrial adaptation [115]. Moreover, glucocorticoids influence TET enzymes and DNMTs, altering the DNA methylation patterns in trophoblasts and decidual cells. This suggests the potential for epigenetic modifications that may affect foetal or placental development.

Recent randomised controlled trial outcomes in reproductive immunological failure groups further underscore safety issues. Women treated with prednisone showed no enhancement in live birth rates and a heightened incidence of biochemical pregnancy loss in these comprehensive multi-centre studies. The results suggest that severe suppression of physiological inflammatory processes may hinder early implantation instead of facilitating it, albeit originating from non-donor populations [77]. The susceptibility of recipients of donor eggs, whose immune systems are already subjected to heightened allogeneic stress, to these adverse consequences remains ambiguous. Glucocorticoids must be judiciously administered in this sensitised context, since pregnancies achieved by donor eggs present an increased risk of maternal problems, such as hypertension, aberrant placentation, and immune-mediated vascular dysfunction. In the absence of safety data pertaining to donor eggs, it is unfeasible to ascertain whether glucocorticoids mitigate these hazards by modulating the immune system or exacerbate them by disrupting critical inflammatory and angiogenic pathways [116].

## 6. Discussion

Our review’s aggregated data highlights a significant paradox in peri-implantation glucocorticoid therapy: although oocyte donation pregnancies display unique biological traits that are suggestive of an increased alloimmune challenge, potentially making immunomodulation attractive, the overall clinical data largely do not show a consistent benefit from empirical glucocorticoid use. Systematic analysis of mechanistic, observational, and randomised studies reveals significant variability in patient selection, immune system assessment, therapy timing, glucocorticoid type, and patient outcomes. This heterogeneity explains, at least partially, the persistent ineffectiveness of glucocorticoids in extensive randomised trials, while sometimes demonstrating efficacy in carefully chosen groups. We compile and contrast the outcomes of all major research categories below to provide you with a clearer understanding of the current state of the field.

OD pregnancies have a much more complex immunological and endometrial environment than autologous IVF. A critical investigation in reproductive immunology is whether the increased allogeneic environment makes OD recipients more amenable to peri-implantation immunomodulation, particularly glucocorticoid treatment [2,35]. The discrepancies become evident when comparing the biological explanation with clinical data: ovarian dysfunction cycles clearly exhibit increased endometrial vulnerability and immunological activation. Nevertheless, no intervention, be it glucocorticoids or other therapies, has consistently shown a clinically verified benefit in improving receptivity [117].

Silvestris et al. show substantial evidence that OD pregnancies signify an immunologically augmented gestational condition [1]. Their thorough research reveals that, compared to autologous IVF, OD pregnancies have increased rates of gestational hypertension, pre-eclampsia, aberrant placentation, and recurrent losses. The authors emphasise that total foetal allogeneicity, defined by both gametes being immunologically alien to the recipient, promotes the development of a trophoblast decidua interface marked by elevated endothelial stress, enhanced NK trophoblast interaction, and augmented antigen presentation. The results support the accepted idea that OD operates as a “semi-transplant” model of pregnancy, where the success of implantation depends on controlled inflammatory signalling and the proliferation of regulatory T-cells.

Pathare et al. provide an alternative perspective by demonstrating that individuals with ovarian dysfunction often have accelerated endometrial ageing [118]. Their examination of stromal fatigue, endometrial senescence, compromised decidualisation, and epigenetic drift offers mechanistic insights into the atypical responses of the endometrium to hormonal or inflammatory stimuli across ovulatory cycles. Pathare et al. contend that a “fragile endometrium,” characterised by increased susceptibility to immunological tone abnormalities, arises from cellular senescence in stromal precursors, elevated production of SASP cytokines, and dysregulated PR-dependent transcription [118]. This explains the diversity in implantation rates in OD pregnancies, despite the use of high-quality embryos.

Although the clinical literature contradicts this notion, these mechanistic findings seem to support the use of glucocorticoids. In the Cochrane evaluation of FET and donor-oocyte endometrial preparation, Glujovsky et al. assessed all available RCTs and found no substantial evidence that steroids improve clinical pregnancy or live birth rates or promote endometrial receptivity [119]. The authors assessed the quality of evidence for steroids as being “very low,” due to methodological errors and an absence of mechanistic stratification. Their results demonstrate a considerable disparity: whereas OD physiology suggests increased immunological sensitivity, therapies aimed at modifying the immune environment have not produced notable clinical improvements.

The prospective research conducted by Tersoglio et al. is among the few studies that only examines OD patients with RIF [120]. Their holistic treatment strategy improved endometrial thickness, normalised leukocyte infiltration patterns, treated chronic endometritis, and eventually elevated live birth rates. The research cannot exclusively attribute enhancements to glucocorticoids, since steroids were just one element of a complex regimen that included hormonal regulation, adjunct treatments, and antibiotics. Zou et al. demonstrated that prednisone only improves reproductive outcomes in RIF patients when chronic endometritis is present and addressed [121]. Tersoglio et al. identified that the primary factor contributing to reproductive enhancement was the rectification of histological issues, particularly the resolution of chronic endometritis [120]. Both studies indicate that steroids may only benefit those with osteoarthritis who have established an inflammatory pathology, rather than the whole osteoarthritis population.

This claim is further validated by a comparison with research on immunological testing. Kwak-Kim et al. and Genest et al. highlight the inherent shortcomings of empirical immunomodulation without diagnostic markers [122,123]. Both evaluations indicate that individuals with OD have a diverse array of autoimmune predispositions, cytokine dysregulation, NK cell activation, and persistent inflammatory endometrial lesions. The benefits of glucocorticoids at the group level are unlikely to be achieved without appropriate stratification techniques, such as autoantibody profiling, NK functional tests, flow cytometry phenotyping, or gene expression profiles. Robertson et al. provide mechanistic caution, indicating that corticosteroid-induced immune suppression may obstruct physiological implantation processes, including EVT invasion, controlled inflammatory signalling (NF-κB, IL-6, and LIF), and uNK-mediated angiogenesis [94]. Administering glucocorticoids universally may exacerbate the risks of endothelial dysfunction and poor placentation, notwithstanding the prevalence of these issues in OD pregnancies.

Despite the frequent use of glucocorticoids in clinical IVF therapy, frequently based on theoretical immunological justifications rather than shown efficacy, the data constantly shows a substantial discrepancy between biological plausibility and clinical outcomes. A thorough examination of randomised trials, meta-analyses, and mechanistic studies reveals that steroids do not improve implantation or live birth rates in unselected IVF populations. Moreover, their indiscriminate use may be harmful [77]. Notwithstanding the inconsistencies in published research about dose, time, glucocorticoid kind, patient selection, and outcomes, a conclusive determination is made: ordinary glucocorticoid administration is ineffectual in the absence of verified immunological disease.

The most comprehensive synthesis is found in Boomsma et al.’s revised Cochrane Review, which included 16 randomised controlled trials, involving over 2200 couples [2]. The results demonstrated that peri-implantation glucocorticoids did not significantly improve live birth rates, continuing pregnancies, clinical pregnancies, miscarriages, or multiple gestations. The evidence quality was assessed as being extremely low to low, mostly due to poor reporting and methodological issues in previous studies. Nevertheless, the impact estimates consistently indicated no substantial treatment advantage, despite these constraints. These findings correspond with the earlier Cochrane analysis by Glujovsky et al., which evaluated endometrial preparation in frozen–thawed and donor-oocyte cycles [119]. In this case, “steroids versus no steroids” did not improve any clinically important outcomes, including live birth, clinical pregnancy, endometrial thickness, miscarriage, or cycle cancellation. When considered together, these two reliable syntheses provide compelling higher-level evidence that the empirical use of glucocorticoids does not improve reproductive success.

Recent datasets included in meta-analyses over the last three years to reevaluate the problem have produced results that are essentially consistent. Lin et al. discovered that the incorporation of glucocorticoids into ovarian stimulation did not alter the frequencies of live births, miscarriages, or implantations [68]. Subgroup and sensitivity analysis revealed that this impact was very fragile, disappearing in certain groups and being greatly affected by study heterogeneity, despite the stated little rise in the clinical pregnancy rate. Likewise, Lv et al.’s meta-analysis revealed a relationship between glucocorticoids and increased clinical pregnancy rates [124]. However, a comparison examination exposes significant methodological flaws that compromise the credibility of their findings. In women who were autoantibody-negative or undertaking their first IVF cycle groups that represent the bulk of standard IVF practice, the beneficial correlation completely dissipated. Only women exhibiting positive immune responses or those having many IVF tries could maintain the advantage, indicating that the reported “positive effect” reflects preexisting immunological disease, rather than a universal improvement in receptivity.

Sun et al.’s multi-centre randomised controlled trial, which randomly allocated 715 patients with precisely defined RIF to receive either 10 mg of prednisone or a placebo, provides the most conclusive and up-to-date data [77]. This experiment is particularly beneficial, since it addresses several issues that were present in previous trials, including insufficient statistical power, inconsistent luteal support, lack of uniform embryo quality, and ambiguous terminology. The definitive findings indicated that prednisone did not increase the live birth rate. Prednisone treatment was linked to a twofold increase in preterm birth rates and a notable escalation in biochemical pregnancy loss, prompting considerable concerns over the safety of empirical steroid usage during early pregnancy. Rather than just indicating “no benefit,” these findings imply that steroids may actively interfere with natural immune trophoblast dynamics. This corroborates the mechanistic apprehensions articulated by Robertson et al., who contended that peri-implantation immune suppression may hinder decidualisation, angiogenesis, and placental development [83].

Previous clinical studies confirm the trend of ineffectiveness. Despite many ideas suggesting that low responders may gain from immune regulation, Bider et al.’s investigation into dexamethasone supplementation in low responders revealed no enhancement in follicular growth, oestradiol levels, maturity profile, or pregnancy rate [125]. Tan et al. established that, despite the significant inflammatory component of OHSS physiology, hydrocortisone and prednisone did not improve fertilisation or implantation, nor did they diminish the incidence of OHSS [126]. Despite limitations due to small sample numbers, these exploratory results support the more substantial current data, suggesting that glucocorticoids provide no benefits to IVF populations without particular immunological disorders.

The conflicting results in glucocorticoid research concerning assisted reproduction highlight essential biological factors governing implantation and endometrial receptivity, transcending simple methodological variations [25]. Implantation is a well-coordinated series of controlled inflammatory activation, decidual transformation, trophoblast invasion, and angiogenic remodelling, rather than a passive or anti-inflammatory event. Immune cells, chemokines, and cytokines provide signals that regulate these activities spatially and temporally [127]. Glucocorticoids may mitigate detrimental inflammation or, if misapplied, inhibit the essential physiological processes required for implantation by extensively obstructing NF-κB, AP-1, STAT pathways, and innate immune mediators. The conflicting results documented in the literature arise from this dualism.

Robertson et al. demonstrate that the initiation of implantation depends on a transient pro-inflammatory increase involving IL-1β, IL-6/LIF, TNF-α, and subsequent STAT3 signalling [83]. This is one of the most plausible mechanical explanations for the lack of benefits in unselected IVF populations. These chemicals recruit immune cells from the vicinity, degrade components of the extracellular matrix, prepare the epithelium for embryo implantation, and initiate the process of early decidualisation. Administration of glucocorticoids at this period precisely obstructs the pathways that are essential for normal embryo attachment. The results of Sun et al.’s comprehensive randomised controlled trial demonstrated that women treated with prednisone for recurrent implantation failure had a higher rate of biochemical pregnancy loss and preterm delivery, with no observable benefit, which closely corresponds with this mechanistic framework [77]. Excessive suppression of the first inflammatory phase may disrupt the adhesion cascade, impede early EVT penetration, and adversely affect placental development, all of which are potential biological consequences.

Glucocorticoids may also impede implantation by modifying the biology of uterine natural killer cells. uNK cells are essential for trophoblast-mediated vascular transformation and the remodelling of spiral arteries, especially the CD56 bright angiogenic fraction [11]. Their signalling requires a coordinated activation of VEGF, PlGF, IL-8, and other pro-angiogenic mediators. Steroids suppress these pathways, causing uNK cells to become quiescent, which is incompatible with the substantial vascular changes that are necessary in the early stages of pregnancy. Silvestris et al. contend that recipients of donor eggs exhibit heightened vulnerability to placental malfunction, hypertensive diseases, and maladaptive vascular remodelling, emphasising the importance of this mechanistic insight [1]. The suppression of angiogenic uNK activity due to steroids may worsen an already precarious compensatory environment in this situation.

Moreover, the biology of decidual stromal cells offers a persuasive molecular framework for clarifying the diverse effects of steroids. Pathare et al. discovered that endometrial ageing, prevalent in women using donated eggs, diminishes the body’s responsiveness to progesterone, alters the activity of FOXO/FOXL2 transcription, increases oxidative stress, and impairs mitochondrial function [118]. Glucocorticoids adversely affect these processes by interfering with progesterone-mediated decidual gene networks, affecting mitochondrial metabolism, and modifying epigenetic markers, including DNA methylation patterns. Consequently, steroids may impede rather than improve endometrial competence in women displaying age-related decidual vulnerability [128].

The immune profiles of patients provide a substantial source of variability. Research demonstrating the advantages of glucocorticoids often involves women with identifiable immunological disorders. Li et al. state that women with unexplained positive autoantibodies receiving glucocorticoid therapy display improved clinical pregnancy and live birth rates, especially when treatment starts before conception [129]. Zou et al. state that individuals with chronic endometritis receiving prednisone and doxycycline have increased rates of implantation and prolonged pregnancy [121]. These results sharply contrast with the neutral effects seen in unselected IVF cohorts, as reported by Boomsma et al., Lin et al., Glujovsky et al., and the prior study by Bider et al. [2,68,119,125]. The answer is clear: glucocorticoids lack a substantial therapeutic target in women whose implantation failure is not due to severe or pathological inflammation. Conversely, they may obstruct critical physiological signals.

The immunobiology of donor egg cycles adds an additional layer of complexity. These pregnancies have increased alloimmune activation and a more significant foetal–maternal antigenic discrepancy. The current research indicates that the processes governing OD implantation are heavily reliant on sustained inflammatory onset, robust uNK angiogenic activity, and preserved decidualisation, notwithstanding the putative benefits of glucocorticoids in this scenario. Silvestris et al. say that OD pregnancies are associated with increased risks of pre-eclampsia, placental abnormalities, and immunologically induced complications: conditions that need targeted immunoregulation instead of nonspecific suppression [1]. Glucocorticoids may unintentionally disrupt these pathways without biomarker guidance.

## 7. Future Direction

Research on peri-transfer glucocorticoids should shift from empirical administration to a precision medicine approach, informed by fundamental processes. Despite compelling evidence that implantation is regulated by distinct inflammatory, genetic, and stromal mechanisms, clinical studies have consistently regarded all patients as immunologically identical. The inconsistency of the present study’s outcomes arises not from variations in therapy but from discrepancies in diagnosis. Rectifying this discrepancy is crucial for the advancement of glucocorticoid research on in vitro fertilisation, particularly for the use of donor oocytes.

A crucial first step is to establish standardised immunophenotyping protocols that differentiate between abnormal inflammation and healthy peri-implantation activity. Substantial evidence exists to substantiate this endeavour. Zou et al. illustrate that a definitive diagnosis is crucial for the effectiveness of steroids in managing chronic endometritis [121]. Li et al. assert that women with autoantibodies have distinct reactions compared to those without them [129]. Sun et al.’s extensive randomised controlled trial showed that treating immunologically unselected recurrent implantation failure patients might exacerbate their condition [77]. These findings indicate the need for validated testing platforms that include cytokine profile panels, Th1/Th2 and Th17/Treg ratios, transcriptome signatures, NK cytotoxicity, and histological markers such as CD138 for chronic endometritis. Clinical studies must use these assessments as eligibility criteria, rather than exploratory outcomes to identify authentic candidates for immunosuppression.

Simultaneously, more study on donor egg cycles is necessary, rather than relying on data drawn from autologous IVF trials. Silvestris et al. contend that OD pregnancies have a unique immunological profile marked by increased susceptibility to compromised placentation, altered complement activity, and heightened alloimmune activation [1]. These biological characteristics suggest that the effects of glucocorticoids may significantly differ from those seen in autologous situations. Therefore, future trials must explicitly include donor egg recipients, classify participants according to immunologic profile and maternal age, and assess outcomes that reflect the specific vulnerabilities linked to ovum donation pregnancies, such as the prevalence of hypertensive disorders, premature placental vascular remodelling, and patterns of angiogenic factors.

Timing and dose that consider the mechanism are further critical objectives. The existing studies demonstrate considerable diversity in steroid type (prednisone, prednisolone, dexamethasone), dose, and duration, with no attempt to synchronise delivery with the immunological stages of implantation. To delineate temporal intervals in which glucocorticoids suppress pathogenic, as opposed to normal, signals, single-cell transcriptomics and spatial proteomics should be used for molecular mapping of the peri-implantation endometrium. This technique may illustrate that short, low-dose regimens do not obstruct EVT invasion and uNK-mediated angiogenesis, or that steroid exposure is only appropriate during pre-transfer inflammatory priming, rather than throughout the adhesion cascade. To avoid replicating the results of Sun et al., which indicated detrimental consequences when therapy exceeds a physiologically appropriate duration, such mechanistic specificity is crucial [77].

The genomic and immunogenetic frameworks must be integrated into a more sophisticated research strategy. No glucocorticoid experiment has included KIR/HLA-C matching as a variable. The interaction between maternal KIR haplotype and foetal HLA-C genotype significantly influences uNK activation and trophoblast invasion. Future studies should classify patients based on activating vs. inhibitory KIR genotypes, HLA-C1/C2 status, and other immune-related loci, since increased uNK cytotoxicity constitutes a mechanistically valid target for glucocorticoids. This approach may identify responder subpopulations that are amenable to targeted immunomodulation, which are distinguished by certain signalling imbalances, such as STAT1 dominance, type I interferon signatures, or compromised LIF/STAT3 activation.

Furthermore, trial design must include the biology of endometrial tissue. Pathare et al. contend that receptivity is hindered by deficiencies in decidualisation, mitochondrial malfunction, and epigenetic ageing, regardless of immune activity [118]. These endometrial phenotypes may aggravate dysfunction or reduce any potential benefits of glucocorticoids. Incorporating markers of decidual senescence, progesterone-resistance signatures, and metabolic dysfunction into screening frameworks will enable researchers to differentiate between implantation failure due to immune system factors and that resulting from stromal or epithelial issues that are unresponsive to glucocorticoids.

## 8. Conclusions

Research demonstrates that glucocorticoids are not a universal adjuvant for improving implantation in assisted reproduction, particularly in donor egg IVF cycles, when the vascular and immunological demands of early pregnancy significantly vary from autologous situations. Mechanistically, inflammatory activation, stromal decidualisation, trophoblast invasion, and uNK-mediated vascular remodelling must interact with precision and timing during implantation. Glucocorticoids disrupt these physiological processes by suppressing NF-κB, AP-1, STAT signalling, and numerous cytokine and chemokine networks, except in instances of pathological inflammation. Thus, the varied results seen in clinical trials do not represent genuine inconsistency. Instead they reflect the unstratified use of a medication whose biological effects are profoundly affected by their environment.

Boomsma et al., Lin et al., Glujovsky et al., and prior smaller studies like Bider et al. demonstrate that steroids provide no benefit to immunologically unselected IVF groups [2,119,125,129]. Sun et al.’s extensive multi-centre randomised controlled trial revealed that random treatment of glucocorticoids might exacerbate outcomes by increasing the likelihood of biochemical pregnancy loss and premature birth [77]. In contrast, minor but consistent advantages in some subgroups, such as women with chronic endometritis or unexplained autoantibodies, suggest that glucocorticoids may only be therapeutically appropriate in the context of certain immunopathologies.

There is less justification for using glucocorticoids in donor egg cycles in the absence of data. Silvestris et al. contend that these pregnancies have increased maternal foetal antigenic disparity, enhanced complement activation, and heightened susceptibility to placental failure [1]. Glucocorticoids may aggravate the distinct vascular and immunological susceptibilities of organ transplant patients by reducing uNK angiogenic activity, hindering EVT invasion, and impairing early placentation, rather than promoting implantation. The lack of donor-egg-specific randomised studies emphasises the difficulties in extrapolating from autologous IVF findings and stresses the need for caution.

All biological and clinical discoveries converge on a singular conclusion: the use of glucocorticoids in reproductive medicine should be grounded on precision, rather than conjecture. Their application should be restricted to clearly defined immune-mediated disorders supported by standardised diagnostic evaluations, including autoantibody testing, cytokine profiling, uNK functional analysis, chronic endometritis assessment, and possibly immunogenetic markers like KIR/HLA-C combinations. Glucocorticoids are unlikely to facilitate implantation without corroborating data and may potentially be harmful.

To advance, we need immunophenotype-guided interventions, organ-specific research frameworks, and mechanistic outcome measures that elucidate the intricate connections between the embryo and the endometrium. Regular peri-transfer glucocorticoid medication for donor egg recipients or the broader IVF population cannot be justified until more information is obtained. The only secure, efficacious, and evidence-supported method to alter the receptivity of the endometrium is by precise reproductive immunology, rather than broad immunosuppression.

## Figures and Tables

**Table 1:** Molecular targets of glucocorticoids at the embryo–endometrium interface.

Biological Compartment	Key Pathways	Effect of Glucocorticoids	Potential Impact
uNK cells	KIR–HLA-C, IFN-γ, VEGF	↓ Cytotoxicity, altered cytokine profile	May normalise hyperactivation but impair angiogenesis
Decidual stromal cells	cAMP-PKA-FOXO1, NF-κB	Stabilises decidual phenotype	Preserves receptivity under inflammatory stress
Dendritic cells	PD-L1, IDO1, CD80/86	Promotes tolerogenic phenotype	Enhances Treg induction
T-cells	Th1/Th17 vs. Treg balance	↓ IFN-γ, ↑ IL-10	Restores immune tolerance
Cytokine signalling	STAT1/STAT3 balance	↓ STAT1 dominance	Favours receptivity programmes
Complement system	C3, C5a	Suppression of activation	Limits collateral tissue damage

**Table 2:** Synthesis of clinical evidence on peri-implantation glucocorticoid therapy according to patient phenotype and immune context.

Patient/Cycle Context	Immune Status	Consistency of Clinical Outcomes	Effect on Live Birth	Interpretative Conclusion
Unselected autologous IVF	Physiological peri-implantation inflammation	Highly consistent across RCTs and meta-analyses	No improvement	Empirical glucocorticoid use ineffective
Recurrent implantation failure (unselected)	Heterogeneous, mostly immune-neutral	Consistent high-quality RCT evidence	No improvement; ↑ adverse outcomes	Potential disruption of physiological implantation
Autoantibody-positive IVF	Systemic immune activation	Moderate consistency (observational + meta-analysis)	Improved	Steroids beneficial only with immune pathology
Chronic endometritis-associated RIF	Local pathological inflammation	Consistent observational evidence	Improved after CE resolution	Adjunctive, pathology-dependent benefit
Oocyte-donation IVF (general)	Heightened alloimmune challenge	Sparse, indirect, extrapolated	No proven benefit	Evidence gap; empirical use unjustified
Oocyte-donation IVF with suspected immune dysregulation	Hypothesised immune imbalance	No dedicated RCTs	Unknown	Candidate population for biomarker-guided trials

**Table 3:** Hypothesised responder phenotypes to peri-transfer glucocorticoids in oocyte-donation IVF.

Subgroup	Immunological Features	Rationale for Steroid Use	Evidence Level
KIR AA + foetal HLA-C2	uNK hyperactivation, IFN-γ excess	Reduce cytotoxic signalling	Hypothesis-driven
ISG-high endometrium	STAT1-dominant transcriptome	Suppress interferon signalling	Translational
Complement overactivation	Elevated C3/C5a	Limit inflammatory damage	Preclinical
M1-polarised macrophages	TNF-α, IL-1β dominance	Shift toward M2 phenotype	Observational
Post-CE inflammatory state	Residual stromal inflammation	Restore decidual balance	Limited clinical
uNK cytotoxic phenotype	PRF1/GZMB high	Normalise angiogenic profile	Single-cell data

## Data Availability

No new data were created or analysed in this study. Data sharing is not applicable to this article.
